# River Ecosystem Response to Prescribed Vegetation Burning on Blanket peatland

**DOI:** 10.1371/journal.pone.0081023

**Published:** 2013-11-21

**Authors:** Lee E. Brown, Kerrylyn Johnston, Sheila M. Palmer, Katie L. Aspray, Joseph Holden

**Affiliations:** School of Geography and water@leeds, University of Leeds, Leeds, United Kingdom; University of Yamanashi, Japan

## Abstract

Catchment-scale land-use change is recognised as a major threat to aquatic biodiversity and ecosystem functioning globally. In the UK uplands rotational vegetation burning is practised widely to boost production of recreational game birds, and while some recent studies have suggested burning can alter river water quality there has been minimal attention paid to effects on aquatic biota. We studied ten rivers across the north of England between March 2010 and October 2011, five of which drained burned catchments and five from unburned catchments. There were significant effects of burning, season and their interaction on river macroinvertebrate communities, with rivers draining burned catchments having significantly lower taxonomic richness and Simpson’s diversity. ANOSIM revealed a significant effect of burning on macroinvertebrate community composition, with typically reduced Ephemeroptera abundance and diversity and greater abundance of Chironomidae and Nemouridae. Grazer and collector-gatherer feeding groups were also significantly less abundant in rivers draining burned catchments. These biotic changes were associated with lower pH and higher Si, Mn, Fe and Al in burned systems. Vegetation burning on peatland therefore has effects beyond the terrestrial part of the system where the management intervention is being practiced. Similar responses of river macroinvertebrate communities have been observed in peatlands disturbed by forestry activity across northern Europe. Finally we found river ecosystem changes similar to those observed in studies of wild and prescribed forest fires across North America and South Africa, illustrating some potentially generic effects of fire on aquatic ecosystems.

## Introduction

Catchment-scale land-use change is recognised as one of the major threats to aquatic biodiversity and ecosystem functioning across the globe [1]. Changes to terrestrial habitats due to urbanisation or development of land for agriculture and forestry alter river flow and thermal regimes, sediment loading, and water chemistry [2,3]. In turn, there can be major changes to the abundance and diversity of many aquatic and riparian organisms, as well as alterations to functional processes such as primary production, respiration and nutrient cycling [2,4]. Such effects may be particularly pronounced in headwater tributaries, where aquatic-terrestrial linkages are strong due to the high density of the river network, and because these systems support high biodiversity owing to the heterogeneity of habitats [5].

Fire can lead to substantial changes in catchment vegetation cover, whether used as a tool in land-use management or when it occurs in an uncontrolled form of wildfire. Research into the effects of catchment-scale fire disturbance on river processes, and river ecosystems in particular, has focused predominantly on the effects of wildfires. Some studies have suggested little or no effect of fire (e.g. [6,7]) but others showed clear post-fire changes in aquatic community composition and diversity [6,8,9]. In contrast to wildfire, prescribed burning of vegetation is practiced worldwide [10,11,12], either to mitigate wildfire effects by producing fire breaks, reducing available natural fuel sources or to promote changes in catchment vegetation structure for food production and to maintain biodiversity. However, while there are concerns about the environmental impacts of these regimes there have been far fewer studies of how aquatic ecosystems respond to prescribed burning (e.g. [13,14,15]). Moreover, almost all of the published studies to date that have examined river ecosystem responses to fire have been undertaken in forested systems [but see 16]. This is despite fires being a common occurrence, both naturally and for management purposes, in landscapes such as prairie, chaparral and temperate moorland [17,18,19].

Upland peat-covered landscapes cover approximately 15% of the UK land area, and in some regions there are large areas subject to prescribed burning. For example, 33 % of the upland peat cover in the Peak District of northern England undergoes regular prescribed burning geared at encouraging red grouse (*Lagopus lagopus scoticus*) production [20]. Vegetation removal (shrubs, predominantly *Calluna* spp.) is undertaken in a controlled manner by burning relatively small patches (typically up to 2000 m^2^) on rotations of between 7 to 25 years depending on local conditions. Over time the practice produces a characteristic mosaic of *Calluna* dominated patches which provide nesting sites for grouse, and recently burned patches with exposed soils and young *Calluna* shoots that provide food for grouse. Burning is undertaken year-on-year (in winter months) with patches typically burned quickly and extinguished by hand before the underlying soils ignite. In contrast, uncontrolled wildfires generally burn hotter, for longer and over much larger areas. While there are considered to be benefits of rotational, prescribed patch-scale burning for red grouse populations, concerns have been raised regarding the environmental effects of manipulating catchment vegetation cover in this manner [21] with calls from stakeholders for more evidence to underpin evidence-based policy developments [22,23,24].

Vegetation removal from peat-dominated catchments has the potential to impact receiving aquatic systems through numerous indirect, delayed effects [19]. First, recently burned areas of land lack vegetation cover and sensitive peat-dominated soils are rendered vulnerable to loosening by freeze-thaw and desiccation and subsequent erosion by wind, rain and overland flow [25]. Transport of eroded soils to rivers may lead to sedimentation and more benthic particulate organic matter, with associated effects on biota [26]. Second, burning can alter the hydrology of soils [27] cf. [28,29], and these effects may lead to changes in river flow at the catchment scale, although this latter issue has not yet been evaluated. Third, burning has been associated with changes to soil and river chemistry both at the plot and catchment scale [27,30,31]. In particular, there is some evidence for changes to dissolved metal concentrations [32]. 

Unburned peatland rivers can contain a relatively rich macroinvertebrate fauna with partial turnover of composition both spatially and temporally linked to changes in river water chemistry, riverbed and suspended sediments and thermal variability [33,34]. Changes in some of these environmental properties in peatland river catchments due to vegetation burning have been associated with differences in aquatic macroinvertebrate community structure/composition but so far for only one study [16]. A loss of mayflies and some stoneflies has been observed in rivers draining burned peatland, with concomitant increases in Chironomidae and Simuliidae abundance, attributed mainly to increases in organic sediment concentrations and deposition in rivers. However, further work is necessary to determine the generality of any upland vegetation burning impacts on aquatic ecosystems across multiple sites and over time.

This study aimed to improve our understanding of whether rotational vegetation burning on blanket peatland is associated with significantly different river macroinvertebrate community structure and composition when compared with unburned systems. The work also examined associations between macroinvertebrates, water quality and benthic organic matter in rivers draining burned and unburned catchments. Based on the findings of earlier studies of peatland management effects on river ecosystems [16,19,34,35] we hypothesised that: (H_1_) there would be significant macroinvertebrate community differences attributed to burning, specifically a reduction in taxonomic richness and diversity in burned rivers; (H_2_) there would be lower abundance of Ephemeroptera, but increased abundance of disturbance tolerant organisms such as Chironomidae, Simuliidae and some Nemouridae, in catchments with vegetation burning [9,36]; (H_3_) differences in macroinvertebrate communities/populations would be associated with greater suspended and deposited sediment and dissolved metal concentrations in burned rivers, and; (H_4_) there would be more detritivorous functional feeding groups (filterers, gatherers) but fewer grazers in burned catchments. Our findings are considered in the context of previous work on peatland river systems, in addition to general literature on catchment disturbance and management effects on peatland river ecosystems.

## Methods

### Study sites

Research was undertaken on ten independent rivers across the north of England between March 2010 and October 2011 (Table 1). Landowners and gamekeepers, Natural England and The National Trust granted permission to access the field sites. Five of these rivers drained peatland with no history of vegetation burning for more than six decades (at Trout Beck, Moss Burn, Green Burn), and likely for at least three to five decades at other sites (hereafter termed *unburned* management). Five rivers drained from catchments where there was a mosaic of contemporary burn patches ranging from <1 to 25 years since burning (hereafter termed *burned* management). Potential study sites were identified as those having 2^nd^ order rivers, a predominant soil cover of blanket peat as mapped by the Soil Survey of England and Wales, with peat depths >1m depth at most sites based on our plot-scale measurements, and catchment areas up to 3.1 km^2^. Selected sites had no confounding forest cover, mining activity, major erosion or artificial drainage, and were selected such that both burned and unburned catchments were distributed amongst the catchment geologies typical of the Pennine hills (Table 1). All sites were grazed by sheep but stocking densities were low, typically <1 ewe per ha. Oakner Clough was impacted by a wildfire in April 2011, therefore data collected in summer and autumn 2011 were excluded from analyses.

**Table 1:** Study site catchment management details, locations and catchment size.

**Management /Site**	**Location**	**WGS84 Lat/Long**	**Catchment area (km^2^)**	**Catchment altitude (m AOD)**	**Geology**
***Burned***					
Bull Clough	Midhope Moor, Peak District	53°28′24.8″N; 1°42′46.2″W	0.7	455-541	Carboniferous and Jurassic sandstone
Great Eggleshope Beck	Teesdale, North Pennines	54°40′59.6″N; 2°04′11.9″W	1.6	480-653	Carboniferous mudstone, sandstone and limestone
Lodgegill Sike	Teesdale, North Pennines	54°40′35.5″N; 2°04′04.1″W	1.2	515-608	Carboniferous mudstone, sandstone and limestone
Rising Clough	Derwent Moors, Peak District	53°23′38.4″N; 1°40′25.0″W	1.8	344-487	Carboniferous gritstone and sandstone
Woo Gill	Nidderdale, Yorkshire Dales	54°12′06.1″N; 1°53′26.3″W	1.0	430-546	Carboniferous and Jurassic mudstone
***Unburned***					
Crowden Little Brook	Longdendale, South Pennines	53°30′51.7″N; 1°53′29.7″W	3.1	355-582	Carboniferous gritstone and sandstone
Green Burn	Teesdale, North Pennines	54°40′40.0″N; 2°21′43.9″W	0.7	548-734	Carboniferous sandstone, limestone and shale
Moss Burn	Teesdale, North Pennines	54°41′19.7″N; 2°23′01.7″W	1.4	560-768	Carboniferous sandstone, limestone and shale
Oakner Clough	Close Moss, South Pennines	53°36′11.1″N; 1°58′03.4″W	1.2	240-451	Carboniferous gritstone and sandstone
Trout Beck	Teesdale, North Pennines	54°40′59.6″N; 2°24′46.0″W	2.8	595-794	Carboniferous sandstone, limestone and shale

Vegetation cover in the unburned catchments was predominantly a mixture of *Eriophorum* spp. (cotton grass), *Calluna vulgaris* (heather) and *Sphagnum* spp. (mosses). At the burned catchments, recent burn patches (<2 years since burning) were predominantly exposed soils with only a small cover of *Sphagnum* and *Calluna* shoots. Older burn patches (>5 years since burning) were dominated by *Calluna* at various stages of growth. 

### Field sampling

Rivers were visited six times each over a period of 20 months, once in spring (March/April), summer (June) and autumn (Sept/Oct) in both 2010 and 2011. For each of the six sample seasons, the ten rivers were visited within an approximately one week period to minimise inter-site differences due to temporal dynamics. Five Surber samples (0.05m^2^, 250µm mesh) for benthic macroinvertebrate larvae were collected randomly from riffle habitat in each river during each of the six visits. Samples were preserved immediately in 70% ethanol and returned to the laboratory for sorting, identification and counting. Macroinvertebrates were identified under a light microscope (x40 magnification) to species level for most taxa (mainly Ephemeroptera, Plecoptera and Trichoptera), with Coleoptera identified mainly to genus, Diptera to Family/subfamily and Oligochaeta to class, using standard UK identification keys [see 37 and references therein]. Benthic particulate organic matter (POM) was retained from each Surber sample, then sorted into fine (FPOM <1mm) and coarse (CPOM >1mm) fractions by sieving, then ashed to determine ash free dry mass (AFDM). CPOM and FPOM data were multiplied to a mass (g) per m^2^.

At each river a suite of 19 river environmental variables was collected on each visit. Water temperature and electrical conductivity were monitored continuously using Campbell Scientific CS547A sensors connected to Campbell Scientific CR1000 dataloggers (15 min resolution) then values corresponding to sampling times were extracted. Water samples (500mL) were collected from each river then passed through a 0.45µm filter in the laboratory before analysing for chloride (Cl), sodium (Na), nitrate (NO_3_), sulphate (SO_4_), aluminium (Al), calcium (Ca), iron (Fe), potassium (K), magnesium (Mg), manganese (Mn), silica (Si) and dissolved organic carbon (DOC). Anions were quantified by ion chromatography (Dionex ICS-3000), cations and metals by ICP-OES (Perkin Elmer 5300DV) and DOC by thermocatalytic oxidation (Analytik Jena Multi NC 2100). These data were used to calculate a sum of acid anions (Cl, NO_3_, SO_4_; ΣAA) and sum of base cations (Ca, K, Mg, Na; ΣBC). pH was measured using a Hach HQ40d portable probe. Suspended sediment (SS) concentration was estimated from the dry weight of sediment retained on filter papers. The full suite of river environmental data was not collected in spring 2010 therefore analysis of macroinvertebrate associations with these data was constrained to the latter five collections.

### Data analysis

For each replicate Surber sample, macroinvertebrate community structure was summarised by calculating (a) log_10_(total abundance +1) per m^2^ (i.e. density), (b) taxonomic richness, and (c) 1-Simpson’s diversity [38]. Additionally, relative abundances were calculated for key macroinvertebrate groups (Ephemeroptera, Plecoptera, Trichoptera, Chironomidae, non-chironomid Diptera and Other taxa [i.e. those not included in the five named groups]) and functional feeding groups (FFGs), with designations for the latter following Moog [39]. For the Chironomidae, we identified sub samples and found only the collector-gatherer subfamilies Diamesinae and Orthocladiinae. Actual abundances of Chironomidae, Simuliidae and Nemouridae were collated for analysis as these groups have been suggested previously as families which typically increase in catchments modified by fire [9,16]. 

Multivariate analysis of variance (MANOVA) in SPSS v19 (IBM SPSS Statistics, New York, USA) was used to test for effects of management, season and their interaction on all macroinvertebrate metrics as well as CPOM and FPOM (fully replicated data). Prior to analysis we confirmed that dependent variables for each river did not display significant temporal auto-correlation, and Pearson correlation coefficients were calculated for all pairs of dependent variables to confirm moderate (0.2-0.7) association [40]. Differences between individual rivers were not assessed with MANOVA as the main focus of the study was on management effects. All datasets were tested for normality (Anderson-Darling test) and homoscedasticity (Levene test) with additional visual observation of residual plots, and log_10_, square root or arcsine transformation where necessary.

One-way Analysis of Similarity (ANOSIM) based on Bray-Curtis dissimilarities was undertaken on macroinvertebrate community composition data to determine if the magnitude of difference *between* burned and unburned peatlands was greater than *within* the two individual land management categories. Analysis was undertaken using the Vegan 2.0-6 package in R [41] with 999 permutations. Non Metric Multidimensional Scaling (NMDS) was also undertaken in the Vegan package to ordinate mean macroinvertebrate abundance data (log_10_ x+1 transformed) for each river/season combination. Bray-Curtis dissimilarities were used and the best two-dimensional solution was retained following up to 200 restarts. River environmental variable vectors were fitted to the solution post-hoc using the envfit procedure with 999 permutations. This approach was preferred over direct ordination approaches such as RDA/CCA because NMDS makes no assumptions about the underlying data structure, and better represents the distances between samples in multivariate ordination space [42].

## Results

**Figure 1:**
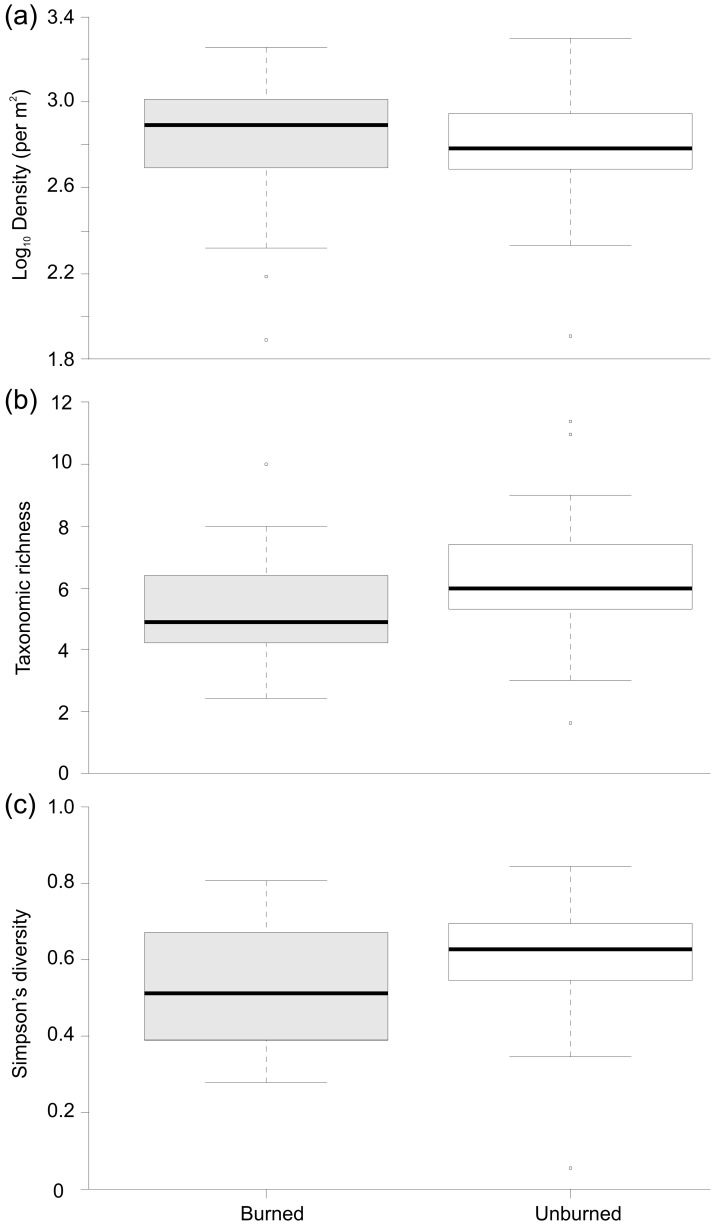
Boxplots summarising (a) Log_10_ Total macroinvertebrate abundance +1, (b) Taxonomic richness and (c) Simpson’s diversity between Burned and Unburned catchments.

Macroinvertebrate abundance ranged from 76 to almost 2000 per m^2^, and we identified 95 taxa from the 300 samples collected as part of the study (Table 2; Figure 1). The maximum richness in individual Surber samples was highest on average in unburned rivers and the maximum number of taxa per sample was 11 (Figure 1). Simpson’s diversity was lower on average, and more variable (IQR), in burned rivers. MANOVA indicated that overall there were significant effects of management (Wilks’ Λ = 0.631, F=10.96, P<0.001, η_p_^2^=0.37), season (Wilks’ Λ = 0.194, F=7.39, P<0.001, η_p_^2^=0.28) and their interaction (Wilks’ Λ = 0.514, F=2.69, P<0.001, η_p_^2^=0.13) on river macroinvertebrate communities. In particular, burned rivers had significantly lower taxonomic richness and Simpson’s diversity but there was no discernible effect on total invertebrate density. ANOSIM revealed a significant effect of burning on the macroinvertebrate community composition (R=0.19; p= 0.001). 

**Table 2:** Descriptive statistics and MANOVA output summaries for macroinvertebrate community metrics and population abundances. Bold values highlight significant differences at p<0.05 and partial eta squared estimates (η_p_^2^) are provided for the determination of statistical effect size.

	**Density (# m^-2^)**	**Richness**	**Simpson’s diversity index**	**% Ephemerop-tera**	**% Plecop-tera**	**% Trichop-tera**	**% Chironomidae**	**% Coleoptera**	**% Diptera**	**% Other**	**% Grazer**	**% Gatherer**	**% Filterer**	**% Shredder**	**% Predator**	**Nemouridae abundance (# m^-2^)**	**Simuliidae abundance (# m^-2^)**	**Chironomidae abundance (# m^-2^)**
**Burned**																		
Mean	796	5	0.53	5.4	49.5	4.4	27.2	1.4	5.4	7.4	1.4	34.2	4.0	52.1	8.3	135	29	259
St. dev	430	2	0.15	13.2	27.6	4.1	25.8	2.6	5.3	15.5	4.9	29.6	4.6	28.8	10.6	161	66	343
Max	1796	10	0.81	57.4	91.1	15.9	77.6	9.9	20.4	70.3	20.5	84.2	20.4	100.0	39.3	616	344	1268
Min	76	2	0.28	0.0	4.5	0.0	0.2	0.0	0.0	0.0	0.0	0.0	0.0	4.5	0.0	0	0	4
**Unburned**																		
Mean	660	6	0.61	25.0	51.5	2.7	9.4	5.3	3.4	8.1	3.2	39.6	6.5	47.1	2.7	45	18	89
St. dev	326	2	0.17	21.5	24.3	2.9	12.7	9.7	3.4	10.5	7.3	25.0	6.4	25.9	3.0	61	56	129
Max	1396	11	0.84	77.7	100.0	9.3	45.7	35.1	12.0	35.1	23.3	87.7	23.9	87.7	12.0	256	292	464
Min	80	2	0.05	0.0	14.7	0.0	0.0	0.0	0.0	0.0	0.0	7.3	0.0	2.2	0.0	0	0	0
**MANOVA results**																		
Management (df=1)	F=0.94; P=0.33; η_p_^2^=0.003	**F=11.9; P=0.001; η_p_^2^=0.04**	**F=11.3; P=0.001; η_p_^2^=0.04**	**F=63.9; P<0.001; η_p_^2^=0.19**	F=0.04; P=0.84; η_p_^2^<0.001	F=1.49; P=0.22; η_p_^2^=0.05	**F=43.7; P<0.001; η_p_^2^=0.14**	**F=12.7; P<0.001; η_p_^2^=0.44**	F=2.04; P=0.16; η_p_^2^=0.07	**F=4.19; P=0.042; η_p_^2^=0.02**	**F=63.2; P<0.001; η_p_^2^=0.19**	F=3.30; P=0.07; η_p_^2^=0.01	**F=5.11; P=0.025; η_p_^2^=0.02**	F=1.61; P=0.21; η_p_^2^=0.006	**F=6.71; P=0.01; η_p_^2^=0.02**	**F=22.5; P<0.001; η_p_^2^=0.08**	F=0.73; P=0.39; η_p_^2^=0.003	**F=26.7; P<0.001; η_p_^2^=0.09**
Season (df=5)	**F=2.52; P=0.03; η_p_^2^=0.04**	**F=7.10; P<0.001; η_p_^2^=0.10**	**F=5.40; P<0.001; η_p_^2^=0.09**	**F=10.82; P<0.001; η_p_^2^=0.16**	**F=47.7; P<0.001; η_p_^2^=0.46**	F=1.82; P=0.11; η_p_^2^=0.03	**F=29.5; P<0.001; η_p_^2^=0.35**	**F=2.73; P=0.02; η_p_^2^=0.05**	**F=2.58; P=0.026; η_p_^2^=0.05**	F=2.09; P=0.07; η_p_^2^=0.04	**F=6.01; P<0.001; η_p_^2^=0.10**	**F=58.8; P<0.001; η_p_^2^=0.52**	**F=2.54; P=0.029; η_p_^2^=0.04**	**F=50.4; P<0.001; η_p_^2^=0.48**	**F=4.24; P=0.001; η_p_^2^=0.07**	**F=3.67; P=0.003; η_p_^2^=0.06**	F=1.94; P=0.09; η_p_^2^=0.03	**F=14.2; P<0.001; η_p_^2^=0.20**
Management* Season (df=5)	F=1.02; P=0.41; η_p_^2^=0.02	**F=3.05; P=0.011; η_p_^2^=0.06**	F=0.98; P=0.43; η_p_^2^=0.02	F=1.85; P=0.10; η_p_^2^=0.03	**F=2.36; P=0.041; η_p_^2^=0.04**	F=1.89; P=0.10; η_p_^2^=0.03	**F=3.69; P=0.003; η_p_^2^=0.06**	F=2.08; P=0.07; η_p_^2^=0.04	F=1.65; P=0.15; η_p_^2^=0.03	F=2.17; P=0.06; η_p_^2^=0.04	**F=6.01; P<0.001; η_p_^2^=0.10**	**F=3.67; P=0.003; η_p_^2^=0.06**	F=1.39; P=0.23; η_p_^2^=0.03	**F=2.92; P=0.014; η_p_^2^=0.05**	**F=2.55; P=0.028; η_p_^2^=0.04**	F=1.44; P=0.21; η_p_^2^=0.02	F=0.78; P=0.57; η_p_^2^=0.01	**F=5.08; P<0.001; η_p_^2^=0.08**

**Figure 2:**
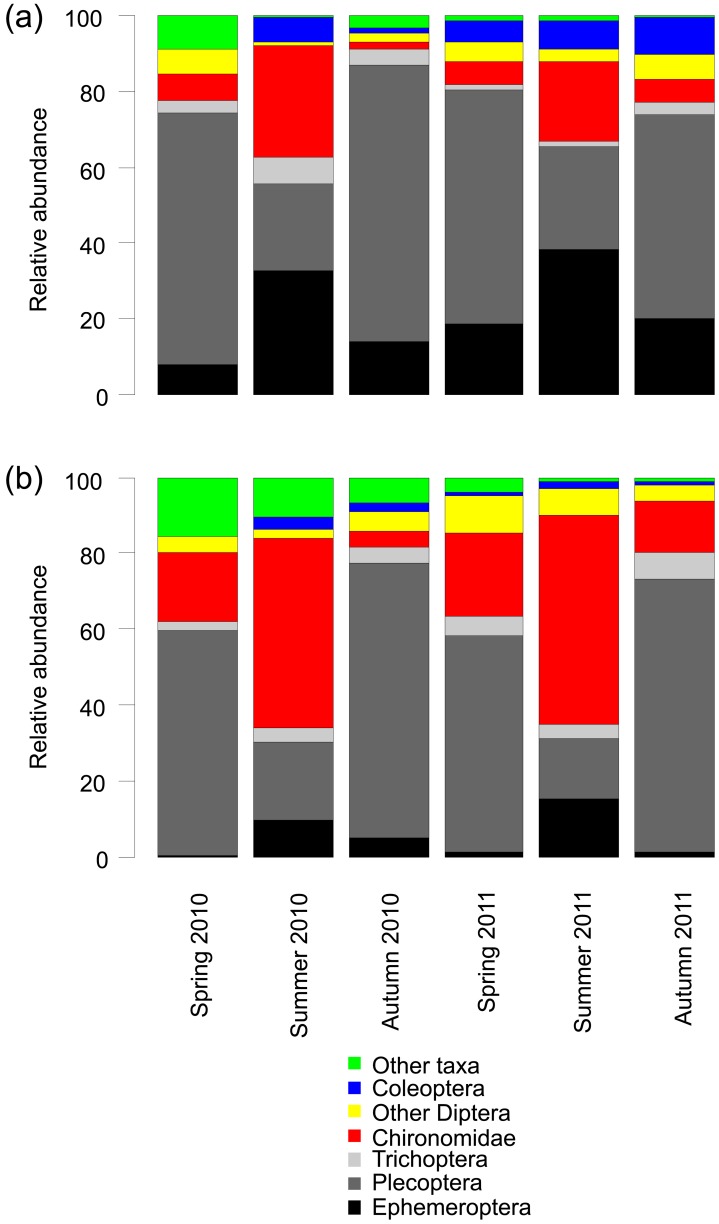
Seasonal changes in the relative abundance of taxonomic groups in rivers draining (a) Unburned and (b) Burned catchments. Data for each season are averages of the five rivers per management category.

**Figure 3:**
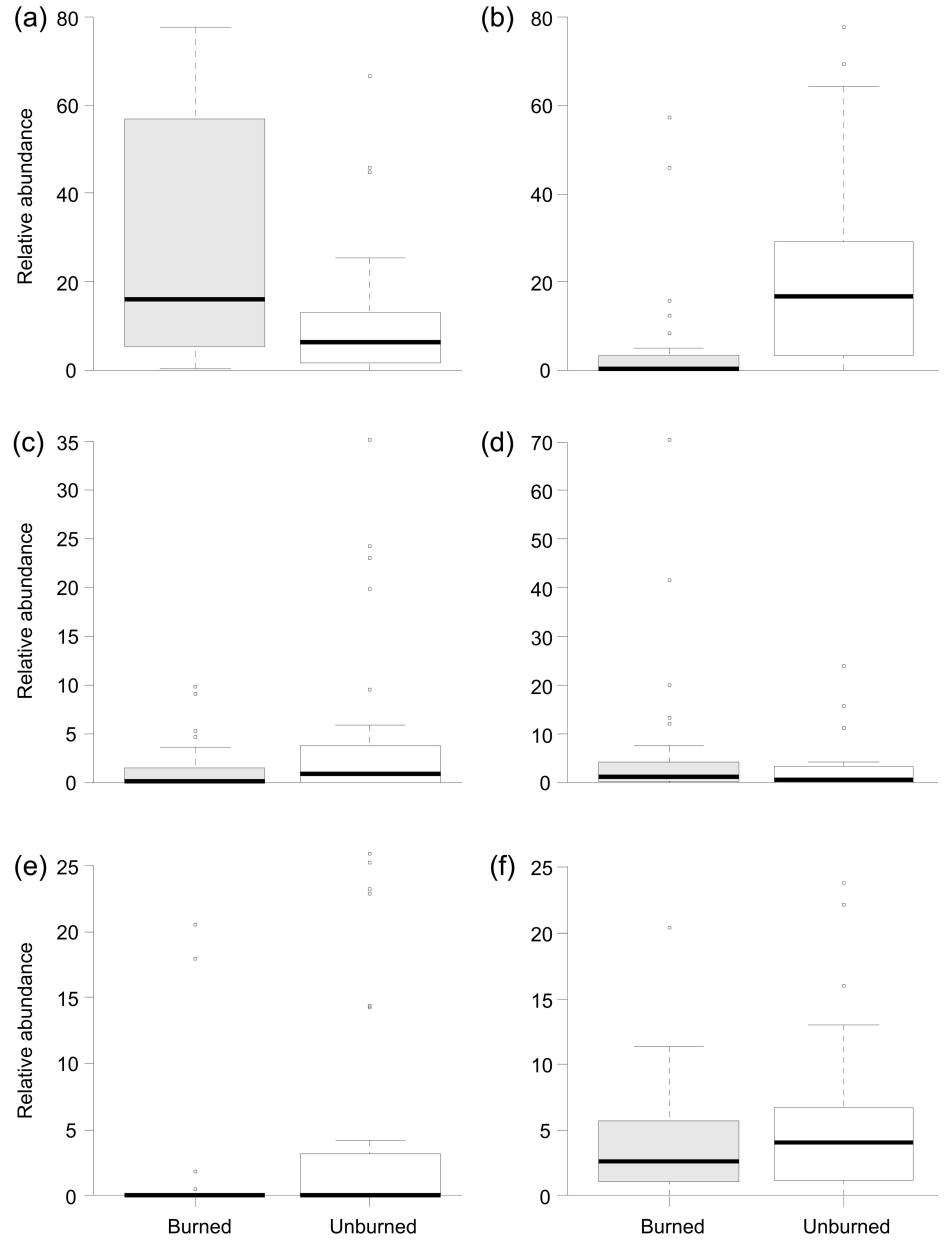
Boxplots summarising relative abundance of (a) Chironomidae, (b) Ephemeroptera, (c) Coleoptera, (d) Other taxa, (e) grazers and (f) filterers between Burned and Unburned catchments.

**Figure 4:**
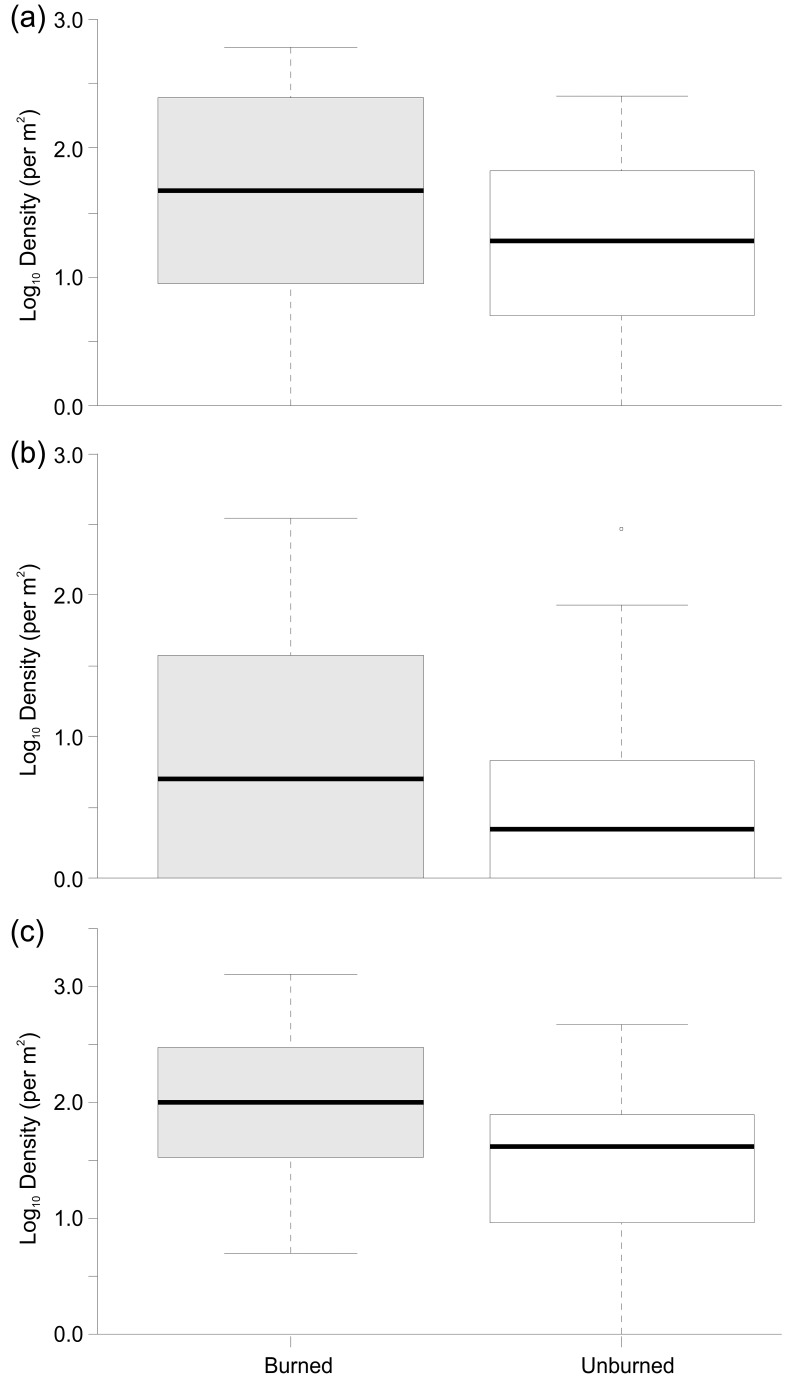
Boxplots summarising Log_10_ (abundance +1) of (a) Nemouridae, (b) Simuliidae and (c) Chironomidae between Burned and Unburned catchments.

At the population level, Plecoptera and Chironomidae were numerically the most abundant groups across all of the samples, although Ephemeroptera were notably abundant in unburned rivers (Figure 2). There were some obvious seasonal variations with Ephemeroptera and Chironomidae accounting for a greater proportion of the communities in summer and Plecoptera being more abundant in autumn and spring samples. Relative abundance of Ephemeroptera, Coleoptera and other taxa were all significantly reduced in burned rivers (Table 2; Figure 2). In contrast, burning was associated with a significant increase in the relative abundance of Chironomidae (Table 2, Figure 3). Actual abundances of Chironomidae and Nemouridae were also significantly elevated in burned rivers (Table 2; Figure 4). For the functional feeding groups, there were significantly higher relative abundances of grazers and filterers in unburned rivers, and more predators in burned rivers. Additionally, there was evidence for a seasonal influence on the magnitude of burning effects on taxonomic richness, actual Chironomidae abundance and the relative abundance of Plecoptera, Chironomidae, grazers, gatherers, shredders and predators. 

**Figure 5:**
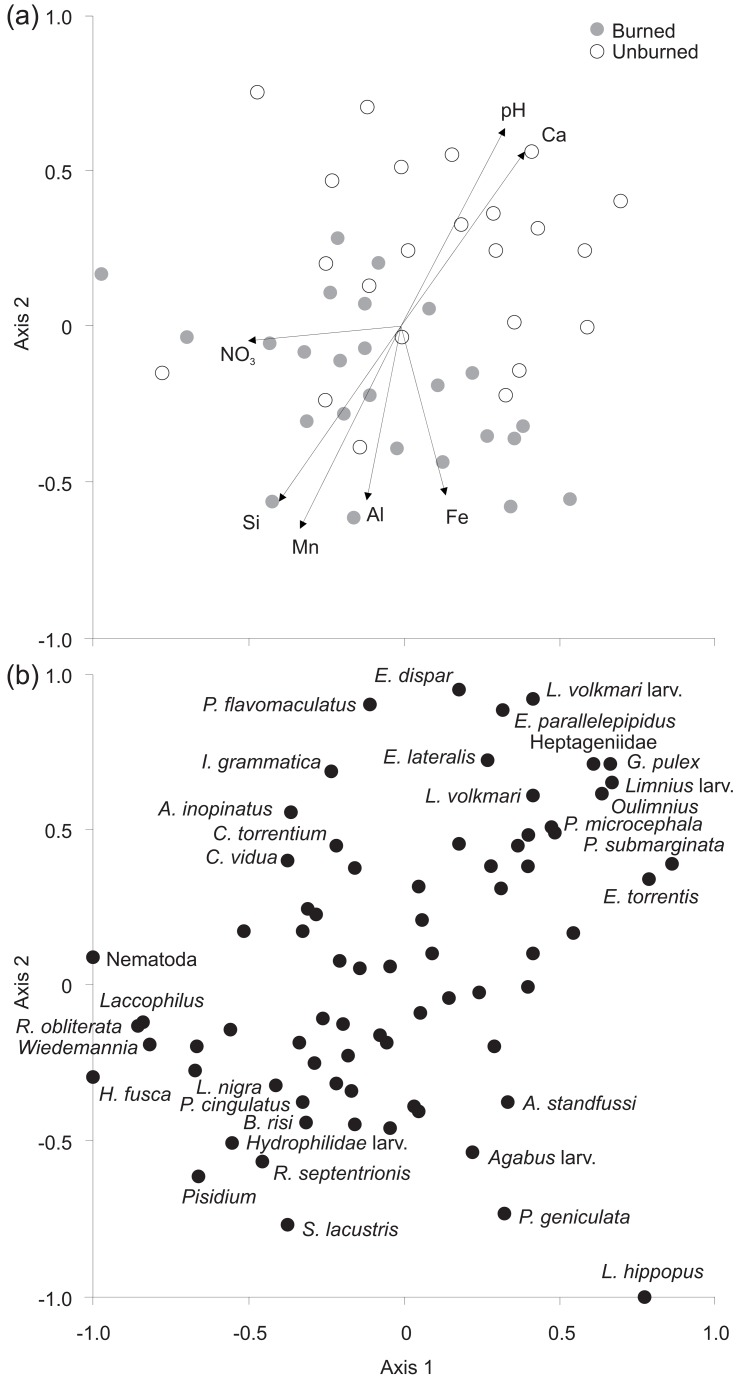
(a) NMDS biplot of samples and significantly correlated environmental variable vectors (pH: R^2^=0.43, p=0.001; NO_3_: R^2^=0.20, p=0.014; Al: R^2^=0.27, p=0.001; Ca: R^2^ = 0.39, p=0.001; Fe: R^2^=0.27, p=0.003; Mn: R^2^=0.46, p=0.001; Si: R^2^=0.42, p=0.001), and (b) taxa.

For CPOM and FPOM, there were overall significant effects of management (Wilks’ Λ = 0.902, F=15.13, P<0.001, η_p_^2^=0.10), season (Wilks’ Λ = 0.825, F=5.60, P<0.001, η_p_^2^=0.10) and their interaction (Wilks’ Λ = 8.98, F=3.08, P=0.001, η_p_^2^=0.05; Table 3). Mean concentrations of FPOM were almost 4x higher, and CPOM almost 3x higher in burned compared to unburned rivers. The NMDS analysis (overall R^2^=0.94, stress=0.03) showed a separation between burned and unburned sites primarily along axis 2 (Figure 5). There was some overlap between the two management categories, primarily because Oakner Clough samples (pre-wildfire) plotted more negatively on axis 2 where most of the burned rivers were plotted. Seven environmental variables (all representing water quality) were correlated significantly with the NMDS output. Calcium and pH were positively associated with unburned sites, whereas burned sites were positively associated with higher Si, Mn, Fe and Al. Elevated NO_3_ concentrations were associated with a small number of outlier samples.

**Table 3:** Descriptive statistics and MANOVA output summaries for benthic organic matter. Bold values highlight significant differences at p<0.05 and partial eta squared estimates (η_p_^2^) are provided for the determination of statistical effect size.

	**FPOM(g m^-2^)**	**CPOM(g m^-2^)**
**Burned**		
Mean	15.0	9.1
St. dev	32.2	12.2
Max	133.0	52.1
Min	0.3	0.7
**Unburned**		
Mean	3.8	3.2
St. dev	5.0	4.9
Max	28.7	49.4
Min	0.07	0.04
**MANOVA results**		
Management (df=1)	**F=18.5; P<0.001; η_p_^2^=0.06**	**F=29.6; P<0.001; η_p_^2^=0.10**
Season (df=5)	**F=6.48; P<0.001; η_p_^2^=0.10**	**F=7.96; P<0.001; η_p_^2^=0.13**
Management*Season (df=5)	**F=3.88; P=0.002; η_p_^2^=0.06**	**F=4.27; P=0.001; η_p_^2^=0.07**

**Figure 6:**
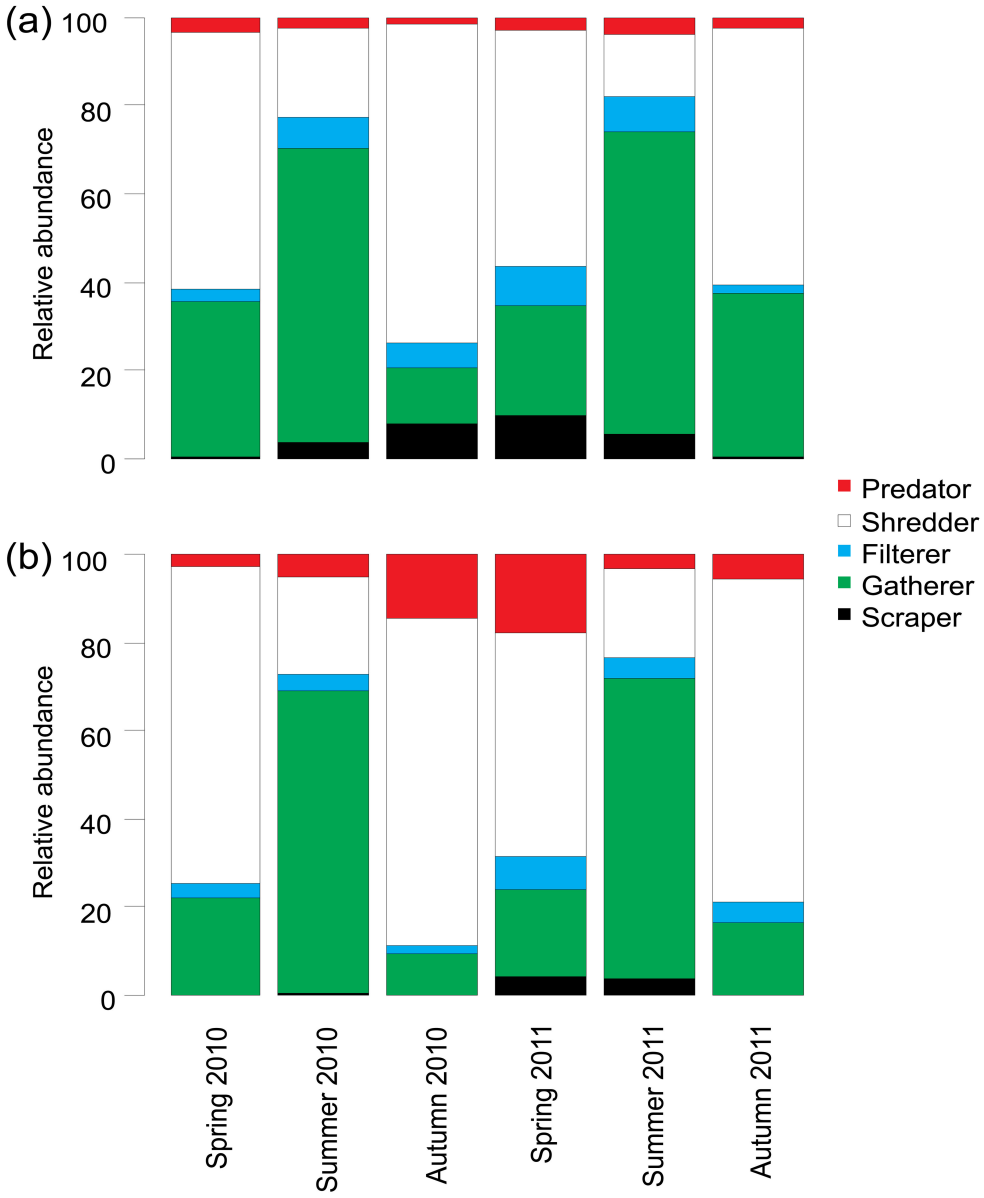
Seasonal changes in the relative abundance of functional feeding groups in rivers draining (a) Unburned and (b) Burned catchments. Data for each season are averages of the five rivers per management category.

All rivers were dominated by detritivorous macroinvertebrates with most being classified as collector gatherers or shredders (Figure 6). Gatherers were relatively more abundant in summer whereas shredders dominated autumn and spring samples. Scrapers were most abundant during the middle of the monitoring period (autumn 2010, spring 2011) in the unburned rivers but in the burned catchments they were found at much lower abundance and were present only irregularly. The MANOVA highlighted that grazer and collector-filterer relative abundance was significantly lower in burned rivers but predator abundance was elevated (Table 2).

## Discussion

### Macroinvertebrate community-level response to prescribed vegetation burning

This study has demonstrated significant decreases in taxonomic richness and Simpson’s diversity in rivers draining burned peatlands, thus we accepted H_1_ that there would be an effect of burning on macroinvertebrates at the community level. These findings are supported by those reported by Ramchunder et al. [16] though we sampled a greater number of rivers over a longer time-scale. The similarity of results in these two studies is a strong indication that there are some common effects of prescribed vegetation burning on upland river ecosystems in the UK. Similar decreases in taxonomic richness have been observed in rivers that are affected by catchment-scale artificial drainage [35], and Ormerod et al. [43] also showed declines in richness where upland coniferous afforestation was undertaken. Reduced richness and diversity are common responses to lower pH and associated changes in water chemistry with acidification [44] as we observed due to prescribed vegetation burning. These findings suggest that land management practices that alter soils and/or vegetation composition in upland catchments might induce some common responses to river macroinvertebrate fauna. 

The difference in macroinvertebrate taxonomic richness observed between burned and unburned systems in our study contrasted with the findings of Minshall et al. [7], Beche et al. [14] and Arkle & Pilliod [13] who found no effect in their studies of wildfire or prescribed forest-fire effects on river ecosystems in the USA. These differences between studies may be a reflection of factors including underlying soil types and terrestrial vegetation structure, as well as prescribed burning regimes being on a patch basis in our peatland study compared to much larger areas in managed US forest systems. However, we found no effect of burning on total macroinvertebrate density, which was similar to observations made in studies of both wildfire and prescribed forest fire effects on rivers in North America [6,7,13]. One potential reason for this lack of response across the entire macroinvertebrate cohort is that increases in the abundance of disturbance tolerant taxa counteract declines and/or losses amongst more sensitive groups. Similar compensatory responses have been noted in peatland rivers affected by artificial drainage [35], and also following wildfire studies in forests of New Mexico [8]. Compensatory responses are common in ecosystems affected by disturbance [45], therefore other pertinent questions that need to be assessed for UK upland river systems affected by fire are whether such effects are seen amongst other biotic groups (e.g. microbes, algae), and if such compensation can buffer effects on higher level ecosystem functions and services.

### Population level response to prescribed vegetation burning

At the population level, we hypothesised (H_2_) lower abundance of Ephemeroptera in burned rivers and this expectation was substantiated with the mean relative abundance of this Order reduced by approximately 20%. Burning was associated with significant decreases in pH and Ca but associated increases in Al, Mn and Fe. These river environment differences were associated with differences in the macroinvertebrates between burned and unburned rivers in the NMDS as predicted for H_3_. Al and pH have long been known to lead to reductions in sensitive mayflies [44,46], with reasons suggested to include Al toxicity, avoidance behaviour and/or reduced growth [47,48,49]. In addition, Mn is a micronutrient which is considered to be toxic to freshwater organisms at elevated levels [50,51], and this may have accounted for some of the macroinvertebrate differences between burned and unburned streams. 

In previous studies of peatland management effects on river ecosystems, mayflies have proven to be good indicators of habitat change [16,35]. In the present study we typically observed *Baetis rhodani* to be the dominant Ephemeroptera taxon in burned rivers, although there were occasional collections of *Leptophlebia*, *Paraleptophlebia* and *Siphlonurus* spp.. *Baetis* spp. have been seen to benefit from wildfire in north American rivers although to a greater extent than we observed [6,52]. These taxa were also present in unburned rivers but overall mayfly assemblages were more diverse and included *Seratella ignita*, various heptagenids and *Ameletus inopinatus* [8]. The Ephemeroptera could therefore be a useful group for rapid, focused assessments of the impacts of prescribed vegetation burning rather than focusing on whole macroinvertebrate community response. Similar suggestions have been made for using Ephemeroptera (plus some other macroinvertebrate groups) to monitor the effects of, and recovery from, upland acidification and forestry activity [44].

In contrast to declines in the abundance of Ephemeroptera, as part of H_2_ we expected that disturbance tolerant organisms such as Chironomidae, Simuliidae and some Nemouridae would increase in abundance in catchments with vegetation burning. Such responses have been seen where forestry activity has altered river ecosystem environments on peatlands in Finland [53], as well as in previous studies of UK uplands [16,35]. However, this hypothesis was not supported for the Simuliidae, perhaps reflecting our finding that suspended sediment concentration did not differ between burned and unburned rivers at the times of sampling, compared with previous peatland studies where these filter-feeders appear to have benefited from more abundant organic particles in the water column. H_2_ was supported for the Chironomidae and Nemouridae though, with both families displaying significantly elevated (almost 3-fold) mean abundance in burned rivers. Chironomidae relative abundance reached an average of 27% (max. 77%) in burned rivers (cf. average 9%, max 46% in unburned), and thus showed a similar pattern to those observed in studies of wildfire in Yellowstone, USA where they typically exceeded 40% in burned sites but were <30% in unburned rivers [7,52]. 

The irruption of Chironomidae seen in our study may have been a consequence of competitive release or the significant increase in availability of benthic POM. Greater benthic POM (both fine and coarse fractions) in burned river systems can be attributed to the removal of the terrestrial vegetation cover and litter layer by fire, which increases the vulnerability of the underlying organic soils to physical erosion [54,55]. The response did not appear to be a consequence of predator release as hypothesised by Ramchunder et al. [16] because we observed a significant (but small) increase in relative abundance of this functional group in burned rivers. More detailed food web studies would help to determine the importance of such species interactions in peatland rivers.

Nemouridae appear to be generally resilient to the effects of fire in river catchments, with our findings supported by studies of wildfire by Vieira et al. [9] in New Mexico, and by Mihuc & Minshall [36] in Yellowstone National Park. It has also been noted previously that Nemouridae abundance increases in rivers affected by prescribed heather burning in the UK uplands [16]. This family of stoneflies is generally characterised by dietary flexibility, univoltine life history, small-body size and an ability to live within fine sediment burrows under conditions of relatively low pH [56]. These traits are clearly beneficial in fire disturbed catchments where there may be changes in basal food availability (e.g. primary producers, POM availability), significant sediment erosion and deposition in rivers as well as alterations to water chemistry.

### Functional feeding group response to prescribed vegetation burning

The fourth hypothesis (H_4_) related to the expectation that functional feeding groups would be different with burning, but our prediction that detritivorous groups (i.e. filterers, gatherers) would have higher abundance in burned catchments was not supported. There were no differences in gatherers while filterer relative abundance was highest in unburned rivers, contrasting with previous studies of peatland burning [16]. However, in this previous study there was no evident pH reduction in burned catchments in contrast to our findings. Lower pH can alter detritus quality [57], so it may be that such a difference in the benthic POM can account for our observations. Further work would be necessary to quantify this because our analyses did not extend beyond quantifying detritus standing stock and nor did it cover water quality variability through time in relation to flow regime. With respect to grazers, our expectation for H_4_, that there would be fewer in burned catchments, was upheld. Most of the grazers in our study were mayflies, therefore the reasons for this finding can seemingly be linked to those discussed above (i.e. acidification and related river environment changes). Grazing can still occur in acid rivers though by generalist detritivore-herbivores such as the Nemouridae which were relatively abundant [58,59]. Further work is needed to examine burning effects on producer biomass/abundance/diversity, before it can be determined whether this is a bottom-up effect linked to depressed algal resources [8,44], or whether differences in the river environment were more important for influencing herbivores directly. 

## Conclusion

This study has provided detailed insights into the spatial and seasonal dynamics of macroinvertebrate communities and their association with environmental variables in river systems managed by prescribed vegetation burning. The results have highlighted that burning is linked to differences in macroinvertebrate metrics at the community and population level. These differences were associated with lower pH and Ca, and increased Al, Fe, Si and Mn in burned peatland rivers. Some of these environmental variables (e.g. Al, Fe) have been observed to be altered in peatland soil solutions affected by burning [32,60] so there appear to be some linkages emerging between soils and rivers at the catchment scale. The surveys detailed in this paper were undertaken at a greater number of sites and repeated over a longer period of time than previous studies [16]. Despite this, the findings were very similar in terms of community and population level differences between burned and unburned rivers, suggesting some generalities of ecosystem response to upland vegetation burning. Both our study and the previous work of Ramchunder et al. [16] have been undertaken on headwater second-order river macroinvertebrate communities. We now need to expand our focus and determine whether prescribed burning effects propagate further downriver, and if other biotic groups show similar responses. The broadly similar responses of macroinvertebrates to those observed by others studying both northern European forested systems, and forests modified by wild and/or prescribed fires in North America, illustrate some potentially generic effects of peatland disturbance and/or fire on aquatic ecosystems regardless of geographical location.
